# Validity of a Self-administered Food Frequency Questionnaire for Assessing Carotenoid Intakes Using Serum Biomarkers in Japan: The Tohoku Medical Megabank Project

**DOI:** 10.2188/jea.JE20250074

**Published:** 2026-02-05

**Authors:** Keiko Murakami, Yudai Yonezawa, Taku Obara, Takahiro Yamashita, Shigenori Suzuki, Junko Ishihara, Ribeka Takachi, Shiori Sugawara, Misato Aizawa, Ippei Takahashi, Mami Ishikuro, Aoi Noda, Hisaaki Kudo, Kazuki Kumada, Masayuki Yamamoto, Shinichi Kuriyama

**Affiliations:** 1 Tohoku Medical Megabank Organization, Tohoku University, Sendai, Japan; 2 Graduate School of Medicine, The University of Tokyo, Tokyo, Japan; 3 Innovation Division, KAGOME Co., Ltd., Tochigi, Japan; 4 Graduate School of Medicine, Tohoku University, Sendai, Japan; 5 Tohoku University Hospital, Sendai, Japan; 6 Graduate School of Environmental Health, Azabu University, Kanagawa, Japan; 7 Nara Women’s University Graduate School of Humanities and Sciences, Nara, Japan; 8 Department of Health and Nutrition, Sendai Shirayuri Women’s College, Sendai, Japan; 9 International Research Institute of Disaster Science, Tohoku University, Sendai, Japan

**Keywords:** carotenoid, food frequency questionnaire, Japan, serum concentration, validity

## Abstract

**Background:**

More research is needed to clarify the health effects of dietary carotenoid intakes, and this requires the use of high-quality assessments of habitual dietary intake. Cohort studies from the Tohoku Medical Megabank Organization included a self-administered food frequency questionnaire (TMM-FFQ) for community-dwelling adults. This study evaluated the validity of carotenoid intakes derived from the TMM-FFQ using serum carotenoid concentrations as the gold standard.

**Methods:**

In Miyagi Prefecture, 88 men and 124 women aged ≥20 years voluntarily agreed to participate in the study and provided completed TMM-FFQs and blood samples in 2019 and 2021. Carotenoids examined included α-carotene, β-carotene, β-cryptoxanthin, and lycopene. Intraclass correlation coefficients (ICCs) were calculated to assess correlations between serum concentrations in 2019 and 2021. Spearman’s rank correlation coefficients were calculated to evaluate the correlations between energy-adjusted dietary carotenoid intakes from the TMM-FFQ in 2021 and the average serum carotenoid concentrations in 2019 and 2021, with correction for the attenuating effect of random within-individual variation.

**Results:**

The ICCs between serum concentrations over the 2 years were >0.50 for all carotenoids. Among men, correlation coefficients were 0.33 for α-carotene, 0.42 for β-carotene, 0.50 for β-cryptoxanthin, and −0.09 for lycopene. Among women, the coefficients were lower than those for men, except for lycopene: 0.11 for α-carotene, 0.23 for β-carotene, 0.21 for β-cryptoxanthin, and 0.28 for lycopene.

**Conclusion:**

The TMM-FFQ demonstrated reasonable validity for assessing intakes of α-carotene, β-carotene, and β-cryptoxanthin among men, but not among women, in the TMM cohort studies when using serum concentrations as the gold standard.

## INTRODUCTION

Epidemiological studies have shown that higher intake of fruits and vegetables is associated with reduced risks of cardiovascular disease, cancer, and mortality.^1^^,^^2^ Because carotenoids are primarily obtained from fruits and vegetables, they may serve as specific biomarkers of fruit and vegetable intake.^3^ Although dietary recommendations for carotenoids should ideally focus on endpoints related to health benefits and well-established mechanisms of action, the link between mechanistic effects and observed health outcomes remains unclear.^4^ Therefore, more research is needed to clarify the health effects of dietary carotenoid intakes, and this requires good quality dietary intake assessment.

Food frequency questionnaires (FFQs) have become one of the primary methods for assessing dietary intake in large-scale epidemiological studies.^5^ Validation of the FFQ is essential, as incorrect and unreliable data may lead to false associations between dietary intakes and health outcomes.^5^^,^^6^ Many studies have used blood concentrations as the gold standard for evaluating the validity of FFQs in assessing carotenoids,^7^^–^^9^ based on the premise that measurement errors in biomarkers are largely independent of those associated with FFQs.^5^^,^^6^ However, the validity of each FFQ should be assessed individually because even minor design changes may influence its performance.^5^^,^^6^ Moreover, validation studies should be conducted in populations similar to those in the main study where the FFQ will be applied.^6^

The Tohoku Medical Megabank (TMM) Project, conducted by Tohoku University Tohoku Medical Megabank Organization (ToMMo) and Iwate Medical University Iwate Tohoku Medical Megabank Organization, has initiated prospective cohort studies in Miyagi and Iwate Prefectures, Japan.^10^^,^^11^ These cohort studies included a self-administered FFQ (TMM-FFQ) for community-dwelling adults.^12^^,^^13^ We previously demonstrated the validity of the TMM-FFQ for assessing carotenoid intake using weighed dietary records as the gold standard in Miyagi Prefecture.^13^ Using multiple validation approaches lends greater credibility to the results.^6^

We, therefore, aimed to evaluate the validity of carotenoid dietary intakes derived from the TMM-FFQ using serum carotenoid concentrations as the gold standard in Miyagi Prefecture.

## METHODS

### Study population

This study was part of the TMM-FFQ validation study, the details of which have been described previously.^12^ The eligibility criteria were individuals aged ≥20 years residing in Miyagi Prefecture, not pregnant, and able to visit one of the community support centers in Sendai, Iwanuma, or Ishinomaki, which were established by ToMMo for voluntary admission-type health assessments of cohort participants.^14^ An optimal sample size for realistic conditions appears to be 100 to 200 persons, which is adequate for likely degrees of validity and allows the exclusion of some participants during validation studies.^5^ The ToMMo study office aimed to recruit 200 persons and finally recruited 228 men and women who voluntarily agreed to participate, meaning they were not selected randomly. All participants provided written informed consent for participation at the study orientation. Among them, 15 participants failed to provide completed TMM-FFQs and serum samples, and 1 participant failed to provide serum samples owing to a busy schedule or other difficulties. A final total of 88 men and 124 women were included in the analyses. The protocol of the present study was reviewed and approved by the ToMMo Research Ethics Committee (2019-4-027) and all other collaborating research institutions.

### FFQ

Participants were requested to complete and submit the TMM-FFQ to the center personnel when they visited the community support center in November 2019 and November 2021.

The TMM-FFQ asks participants about their usual consumption of 139 food and drink items over the previous year.^12^^,^^13^ The frequency categories for food items are “constitutionally unable to eat it” and nine categories ranging from “<once/month or never” to “≥7 times/day.” Portion sizes for food items are categorized as 50% smaller than standard, standard, or 50% larger. The frequency categories for drink items are “constitutionally unable to drink it” and nine categories ranging from “<once/week or never” to “≥10 cups/day.” Some participants completed the FFQ with assistance from family members who typically prepared their meals. If the staff identified missing answers or logical errors in the FFQ responses, they asked participants to provide that information again.

The major carotenoids found in foods—and the most studied in relation to health—are α-carotene, β-carotene, β-cryptoxanthin, lycopene, lutein, and zeaxanthin.^15^ Intakes of energy, α-carotene, β-carotene, and β-cryptoxanthin were calculated using the Standard Tables of Food Composition in Japan 2010.^16^ Lycopene intakes were calculated using a specially developed food composition table for carotenoids,^8^^,^^17^ because lycopene is not included in the Standard Tables of Food Composition in Japan 2010.^16^ Intakes of lutein and zeaxanthin were not calculated because the corresponding food composition tables were unavailable. Intakes from dietary supplements were not included in calculations for the FFQ because a dietary supplement database was not available.

Information on height, weight, smoking status, and alcohol drinking were collected using a self-administered questionnaire integrated with the FFQ.

### Serum concentrations

A total of 20 mL of blood (9 mL in a tube for serum, 4 mL in a tube for serum, and 7 mL in an EDTA2Na tube) was collected by venipuncture from participants twice, approximately 1 week after they completed the FFQ; namely, November 2019 and November 2021. We had initially planned to collect blood samples in November 2019 and in November 2020, but we had to change this plan because of the coronavirus disease 2019 pandemic. Participants were asked to fast for at least 4 hours before blood collection.

Tubes containing 9 mL of blood were centrifuged at 1700 × g for 10 minutes at 4°C at the ToMMo biobank within the day of blood collection to obtain serum.^18^ The serum was divided into four tubes (each 700 µL of serum) and stored at −80°C at the ToMMo biobank until being sent to KAGOME Co., Ltd. (Nagoya, Japan). Serum concentrations of α-carotene, β-carotene, β-cryptoxanthin, and lycopene were determined at KAGOME Co., Ltd. using high performance liquid chromatography, the details of which have been described previously.^19^ Serum concentrations of retinol and α-tocopherol were also measured but the validity of the TMM-FFQ for them were not examined because serum retinol concentrations have only limited interpretability as measures of dietary intake among well-nourished populations and serum α-tocopherol concentrations may not be good predictors of the diet source only.^3^^,^^20^^,^^21^

### Statistical analysis

Intraclass correlation coefficients (ICCs) were calculated to assess the correlation between serum concentrations measured in 2019 and 2021. To evaluate the validity of the FFQ, Spearman’s rank correlation coefficients (CCs) were calculated between dietary intakes from the FFQ and serum concentrations of α-carotene, β-carotene, β-cryptoxanthin, and lycopene. Dietary intakes from the FFQ in 2021 and the average serum concentrations from blood samples collected in 2019 and 2021 were used because collecting biomarker samples both at the beginning and end of the study period is ideal in validation studies.^5^ All dietary intakes from the FFQ were adjusted for energy intake via residual methods,^22^ using linear regression analyses after logarithmic transformation. CCs were then corrected for the attenuating effect of random within-individual variation.^23^ It is almost impossible to set cutoff values for validation studies because the important aspects of validity vary depending on the aim of the FFQ.^6^ However, lower correlations, such as 0.3 or 0.4, will attenuate associations.^6^ One review of FFQs validated in Japan using dietary records as the gold standard applied the following cutoff values: CCs ≥0.60 were categorized as strong, 0.40–0.59 moderate, 0.30–0.39 fair, and <0.30 poor.^24^ Considering that the correlation between a dietary assessment method and a concentration biomarker can be taken as an estimate of the lower limit of validity,^3^ we assumed CC ≥0.30 as a reasonable validity in the present study. The validity of the categorization was measured by calculating the number of participants classified into the same, adjacent, and extreme categories by cross-classification according to both quintiles using the FFQ and the serum concentrations.^5^^,^^6^ Sensitivity analyses were conducted excluding current smokers because smoking is associated with lower serum carotenoid concentrations.^3^^,^^4^ The validity based on the FFQ in 2019 and the serum concentrations in 2019 was also examined, considering the possibility of overestimation due to multiple responses to the same FFQ within the same population.

All analyses were conducted for men and women separately using SAS version 9.4 software (SAS Institute Inc., Cary, NC, USA).

## RESULTS

Table 1 shows the characteristics of the participants. The ≥70 years age group contained the highest percentage of men, and the <40 years age group contained the highest percentage of women. The percentages of participants with a body mass index ≥25.0 kg/m^2^ were >35% of men and >20% of women. Approximately 20% of men and approximately 1% of women were current smokers. Approximately 70% of men and approximately 40% of women were current drinkers. Among men, the ICCs for serum concentrations between 2019 and 2021 were 0.82 for α-carotene, 0.84 for β-carotene, 0.71 for β-cryptoxanthin, and 0.57 for lycopene. Among women, the corresponding ICCs were 0.56 for α-carotene, 0.68 for β-carotene, 0.75 for β-cryptoxanthin, and 0.54 for lycopene.

**Table 1.** Characteristics of participants

	Men		Women	
	*n* (%)		*n* (%)	
Age				
<40 years	17 (19.3)		30 (24.2)	
40–49 years	17 (19.3)		22 (17.7)	
50–59 years	13 (14.8)		22 (17.7)	
60–69 years	11 (12.5)		25 (20.2)	
≥70 years	30 (34.1)		25 (20.2)	
Body mass index				
<18.5 kg/m^2^	2 (2.3)		10 (8.1)	
18.5–24.9 kg/m^2^	54 (61.4)		85 (68.5)	
≥25.0 kg/m^2^	32 (36.3)		28 (22.6)	
Missing	0 (0.0)		1 (0.8)	
Smoking status				
Current smoker	17 (19.3)		1 (0.8)	
Past smoker	44 (50.0)		25 (20.2)	
Never smoker	27 (30.7)		98 (79.0)	
Alcohol drinking				
Current drinker	64 (72.7)		50 (40.3)	
Past drinker	3 (3.4)		1 (0.8)	
Never drinker	17 (19.3)		58 (46.8)	
Constitutionally never drinker	4 (4.6)		15 (12.1)	

Table 2 shows carotenoid intakes from the TMM-FFQ, serum carotenoid concentrations, their correlation coefficients, and comparisons based on cross-classification by quintiles. For men, the crude Spearman’s CCs were 0.33 for α-carotene, 0.41 for β-carotene, 0.45 for β-cryptoxanthin, and −0.03 for lycopene. After energy adjustment and correction for attenuation, the CCs remained largely unchanged at 0.33, 0.42, 0.50, and −0.09, respectively. The proportion of men classified into the same or adjacent quintile categories was highest for β-cryptoxanthin (73.9%) and lowest for lycopene (48.9%). The highest proportion of extreme categories was observed for lycopene (11.4%), and the lowest for β-carotene (1.1%). For women, the crude CCs were 0.17 for α-carotene, 0.25 for β-carotene, 0.18 for β-cryptoxanthin, and 0.22 for lycopene. After energy adjustment and correction for attenuation, the CCs were 0.11, 0.23, 0.21, and 0.28, respectively. The proportion of women classified into the same or adjacent categories was highest for β-carotene (62.9%) and lowest for β-cryptoxanthin (53.2%). The highest proportion of extreme categories was for α-carotene (7.3%) and the lowest for lycopene (3.2%).

**Table 2.** Carotenoid intakes from the TMM-FFQ and serum carotenoid concentrations, their correlation coefficients, and comparisons based on cross-classification by quintiles

	TMM-FFQ		Serum concentration^a^		Correlation coefficients		Cross-classification^c^		
			
	Mean (SD)		Mean (SD)		Crude	Energy-adjusted and deattenuated^b^	Same category	Same and adjacent category	Extreme category
	µg		µg/mL				%	%	%
Men (*n* = 88)									
α-carotene	485 (558)		0.12 (0.10)		0.33	0.33	22.7	63.6	4.6
β-carotene	2,518 (2,249)		0.34 (0.34)		0.41	0.42	33.0	62.5	1.1
β-cryptoxanthin	672 (1,165)		0.16 (0.11)		0.45	0.50	29.5	73.9	2.3
Lycopene	1,573 (3,233)		0.23 (0.11)		−0.03	−0.09	19.3	48.9	11.4
Women (*n* = 124)									
α-carotene	522 (582)		0.18 (0.11)		0.17	0.11	23.4	54.1	7.3
β-carotene	2,910 (2,717)		0.57 (0.46)		0.25	0.23	29.8	62.9	4.8
β-cryptoxanthin	685 (936)		0.23 (0.19)		0.18	0.21	22.6	53.2	4.0
Lycopene	2,621 (4,252)		0.27 (0.11)		0.22	0.28	21.0	61.3	3.2

SD, standard deviation; TMM-FFQ, Tohoku Medical Megabank food frequency questionnaire.

^a^Mean values of serum concentrations in 2019 and 2021.

^b^Spearman’s rank correlation coefficients based on energy-adjusted values and expressed as deattenuated CC. Deattenuated CCx = observed CCx * SQRT (1 + λx/n), where λx is the ratio of within-individual to between-individual variance for carotenoid x, and n is the number of serum concentrations.

^c^Percentages were calculated according to cross-classification by quintiles based on energy-adjusted dietary intakes from the FFQ and serum concentrations.

Similar results were obtained after excluding current smokers (eTable 1). Correlations between intakes from the FFQ in 2019 and the serum concentrations in 2019 did not show the possibility of overestimation due to multiple responses to the same FFQ (eTable 2).

## DISCUSSION

The present study examined the validity of carotenoid dietary intakes derived from the TMM-FFQ using serum carotenoid concentrations as the gold standard. Serum carotenoid concentrations showed high reproducibility over the 2-year period. Among men, the validity of the TMM-FFQ was reasonable for α-carotene, β-carotene, and β-cryptoxanthin, but not for lycopene. The energy-adjusted and deattenuated CCs were 0.33 for α-carotene, 0.42 for β-carotene, 0.50 for β-cryptoxanthin, and −0.09 for lycopene. Among women, the energy-adjusted and deattenuated CCs were lower than those for men—except for lycopene—with values of 0.11 for α-carotene, 0.23 for β-carotene, 0.21 for β-cryptoxanthin, and 0.28 for lycopene.

The validity was mostly reasonable for carotenoids, except for lycopene, among men in the present study. One meta-analysis reported that weighted mean correlations between carotenoid intakes from FFQs and plasma carotenoids were 0.34 for α-carotene, 0.27 for β-carotene, and 0.39 for cryptoxanthin.^7^ One FFQ validation study of Japanese adults from the Japan Public Health Center-based Prospective Study (JPHC-FFQ) reported that the CCs between dietary intake from the FFQ and serum concentrations were 0.34 for α-carotene and 0.18 for β-carotene among men, and 0.30 for α-carotene and 0.03 for β-carotene among women.^8^ The TMM-FFQ was developed by modifying some of the food items from the JPHC-FFQ and adding the response option “constitutionally unable to eat or drink it”.^12^^,^^13^^,^^25^^–^^27^ The present findings suggest that the validity of α-carotene intakes from the TMM-FFQ among men was retained even after some modifications. The CCs for β-carotene were comparatively higher in the present study than those reported in other studies.^7^^,^^8^ FFQs may perform differently in different populations and cultures.^5^^,^^6^ The above-mentioned JPHC-FFQ validation study was conducted also in Japan, but it differed from the present validation study in study area and period.^8^ These differences may have affected the different validity of β-carotene because dietary carotenoid intake shows high variations both within and between participants in different populations.^15^ There is a particular need for a satisfactory measure of β-carotene, as it is the most widely distributed and the most important provitamin A carotenoid.^15^

CCs for carotenoids, except lycopene, were higher among men than among women. These sex differences were also observed for α-carotene and β-carotene in the JPHC-FFQ validation study.^8^ However, one meta-analysis reported the opposite results: CCs for carotenoids except lycopene were lower among men than among women.^7^ Another review reported that women, on average, consumed higher amounts of carotenoids and had higher blood concentrations than men did, partly accounting for the higher correlations between intake and plasma concentrations for women than for men.^4^ We previously found that correlations between carotenoid intakes from the TMM-FFQ and those from 12-day weighed food records were higher among men than among women; the CCs were 0.46 for α-carotene, 0.60 for β-carotene, 0.63 for β-cryptoxanthin, and 0.39 for lycopene among men, and 0.46 for α-carotene, 0.47 for β-carotene, 0.31 for β-cryptoxanthin, and 0.51 for lycopene among women.^13^ The JPHC-FFQ validation study showed that the cumulative percentage for the 20 foods that contributed most to the intake of each nutrient in dietary records was slightly lower among women than among men for most of the nutrients examined.^28^ The higher correlations between carotenoid intake and serum concentration among men than among women in the present study could be explained by the greater variety of food consumption among women in Japan, and therefore the TMM-FFQ may not include sufficient food items which contain carotenoids among women.

CCs were low for lycopene, especially among men. One meta-analysis reported the lower validity for lycopene compared with other carotenoids.^7^ The JPHC-FFQ validation study also showed low validity for lycopene; the CCs were 0.26 among men and 0.10 among women.^8^ Lycopene is present in large amounts in tomatoes and their products, such as ketchup and juices, as well as in watermelons and pink grapefruits.^15^ One possible explanation for this lower validity is that the portion size of tomatoes assessed in FFQs is inadequate.^8^ It may also have been difficult to estimate lycopene intakes in the present study because the lycopene database was quotation from the United States carotenoid database.^17^ Food composition tables are resources that provide detailed information on the nutritional composition of foods, and they sometimes include foods of importance for specific populations.^15^ However, the carotenoid contents reported in the Standard Tables of Food Composition in Japan are limited to provitamin A carotenoids, such as α-carotene, β-carotene, and β-cryptoxanthin.^16^ For example, in addition to tomatoes and their products, a high level of lycopene was found in the Kintoki carrot, which is cultivated mainly in Japan and is uncommon in other countries.^29^ Lycopene databases for the Japanese population are needed.

A key strength of the present study is the use of serum biomarkers of carotenoids as the gold standard because errors for biomarkers and FFQs can be assumed to be uncorrelated.^3^ Because assessing nutritional biomarkers is almost infeasible in large-scale epidemiological studies, FFQs should be validated against biomarkers in a representative sample of the population to which they are administered.^6^ The present study evaluated the validity of the TMM-FFQ against serum biomarkers in the population residing in the area where the TMM cohort studies are being conducted. Blood carotenoid concentrations can be influenced by cooking method used when preparing carotenoid rich foods.^7^ The TMM-FFQ items include the method used most frequently when cooking vegetables. There is ongoing debate about whether a single blood measurement can reliably reflect serum biomarker concentrations because day-to-day variation in dietary intake can lead to fluctuations in biochemical indicators.^5^ In light of this, we used the average serum concentrations from blood samples collected in 2019 and 2021 and applied statistical adjustments to account for random within-individual variation. This could increase confidence in the findings from the present study.

The present study has several limitations. First, serum carotenoid concentrations may be influenced by factors beyond diet. Individual differences in nutrient absorption and metabolism can lead to variation in biomarker levels,^5^ meaning that correlations between concentration biomarkers and dietary intake estimates are expected to be well below 1.0, even when intake is accurately assessed.^3^ Indeed, the CCs observed in the present study were lower than those previously reported between carotenoid intakes from the TMM-FFQ and 12-day weighed food records.^13^ Because the correlation between a dietary assessment method and a concentration biomarker can be taken as an estimate of the lower limit of validity, the absence of a correlation should not be interpreted as definitive evidence that the FFQ is invalid.^3^ Second, the time frames for FFQ data and serum concentration data differed, as is often the case in validation studies. The TMM-FFQ asked participants to report their usual intake over the previous year, whereas the serum concentrations likely reflect more recent dietary exposure. Nevertheless, the long-term reproducibility of carotenoid biomarkers compares favorably with that of other nutrient biomarkers.^3^ In the present study, the high ICCs between serum carotenoid concentrations in 2019 and 2021, along with the moderate correlations between FFQ-estimated intakes and serum concentrations for carotenoids (except lycopene), support the reliability of the findings despite the mismatch in time frames. Finally, it remains unclear how many food items among those used in the TMM-FFQ correspond to dietary source of lycopene in the Japanese and how many of those were assigned lycopene values due to the limited access to the calculation program.

In conclusion, this study demonstrated the reasonable validity of the TMM-FFQ for assessing α-carotene, β-carotene, and β-cryptoxanthin intakes—but not lycopene—among men. These findings suggest that the TMM-FFQ is at least suitable for estimating intakes of α-carotene, β-carotene, and β-cryptoxanthin among men in the TMM cohort studies, although not among women. However, the absence of significant correlations should not automatically be taken as evidence of invalidity because the correlation between an FFQ and a concentration biomarker can be taken as an estimate of the lower limit of validity.

## SUPPLEMENTARY MATERIAL

eTable 1. Carotenoid intakes from the TMM-FFQ and serum carotenoid concentrations, their correlation coefficients, and comparisons based on cross-classification by quintiles, excluding current smokers

eTable 2. Carotenoid intakes from the TMM-FFQ in 2019 and serum carotenoid concentrations in 2019, their correlation coefficients, and comparisons based on cross-classification by quintiles
