# Supplementary material for: Validity of a Self-administered Food Frequency Questionnaire for Assessing Carotenoid Intakes Using Serum Biomarkers in Japan: The Tohoku Medical Megabank Project
Source: J Epidemiol. 2026 Feb 5;36(2):67–72. doi: 10.2188/jea.JE20250074 (PMC12783503; doi:10.2188/jea.JE20250074)
Supplement: Supplementary file 1 [file je-36-067-s001.pdf]

eTable 1. Carotenoid intakes from the TMM-FFQ and serum carotenoid concentrations, their correlation coefficients, and comparisons based on cross-classification by quintiles, excluding current smokers

	TMM-FFQ	Serum concentration[a]	Correlation coefficients		Cross-classification[c]		
	Mean (SD) µg	Mean (SD) µg/mL	Crude	Energy-adjusted and deattenuated[b]	Same category %	Same and adjacent category %	Extreme category %
Men (n=71)							
α-carotene	534 (599)	0.13 (0.11)	0.22	0.22	23.9	56.3	5.6
β-carotene	2,743 (2,361)	0.38 (0.37)	0.35	0.36	29.6	62.0	0.0
β-cryptoxanthin	725 (1,266)	0.17 (0.11)	0.46	0.53	35.2	76.1	1.4
Lycopene	1,734 (3,535)	0.23 (0.12)	0.005	−0.04	21.1	50.7	9.9
Women (n=123)							
α-carotene	521 (584)	0.18 (0.11)	0.17	0.11	24.4	56.1	6.5
β-carotene	2,909 (2,729)	0.57 (0.46)	0.25	0.24	29.3	64.2	4.9
β-cryptoxanthin	690 (938)	0.24 (0.19)	0.16	0.20	22.8	53.7	4.9
Lycopene	2,641 (4,263)	0.27 (0.11)	0.21	0.27	20.3	61.0	4.9

SD, standard deviation; TMM-FFQ, Tohoku Medical Megabank food frequency questionnaire.

[a]Mean values of serum concentrations in 2019 and 2021.

[b]Spearman's rank correlation coefficients based on energy-adjusted values and expressed as deattenuated CC. Deattenuated CCx = observed CCx * SQRT (1 + λx/n), where λx is the ratio of within-individual to between-individual variance for carotenoid x, and n is the number of serum concentrations.

[c]Percentages were calculated according to cross-classification by quintiles based on energy-adjusted dietary intakes from the FFQ and serum concentrations.

eTable 2. Carotenoid intakes from the TMM-FFQ in 2019 and serum carotenoid concentrations in 2019, their correlation coefficients, and comparisons based on cross-classification by quintiles

	TMM-FFQ in 2019		Serum concentration in 2019		Correlation coefficients		Cross-classification[b]		
	Mean (SD)		Mean (SD)		Crude	Energy-adjusted[a]	Same category	Same and adjacent category	Extreme category
	µg		µg/mL				%	%	%
Men (n=88)									
α-carotene	461	(363)	0.12	(0.12)	0.29	0.34	28.4	68.2	3.4
β-carotene	2,459	(1,854)	0.31	(0.32)	0.40	0.46	28.4	75.0	3.4
β-cryptoxanthin	621	(812)	0.15	(0.09)	0.29	0.33	26.1	69.3	3.4
Lycopene	2,032	(3,654)	0.22	(0.12)	−0.06	−0.05	21.6	50.0	4.6
Women (n=124)									
α-carotene	566	(561)	0.17	(0.11)	0.21	0.20	30.7	63.7	4.8
β-carotene	3,088	(2,290)	0.50	(0.41)	0.34	0.36	24.2	59.7	0.8
β-cryptoxanthin	776	(1,672)	0.23	(0.16)	0.36	0.37	32.3	67.0	3.2
Lycopene	3,939	(14,050)	0.26	(0.13)	0.18	0.22	25.8	58.9	4.0

SD, standard deviation; TMM-FFQ, Tohoku Medical Megabank food frequency questionnaire.

[a]Spearman's rank correlation coefficients between energy-adjusted intakes from the TMM-FFQ in 2019 and serum concentrations in 2019.

[b]Percentages were calculated according to cross-classification by quintiles based on energy-adjusted dietary intakes from the FFQ and serum concentrations.
